# Supplementary material for: The interactome of intact mitochondria by cross-linking mass spectrometry provides evidence for coexisting respiratory supercomplexes
Source: Mol Cell Proteomics. 2017 Dec 8;17(2):216–32. doi: 10.1074/mcp.RA117.000470 (PMC5795388; doi:10.1074/mcp.RA117.000470)
Supplement: Supplemental Data [file supp_RA117.000470_133922_0_supp_23978_fzffwf.zip › spectra_annotation/mito_DR_spectra_annotation/185-1-5-1-17-1.pdf]

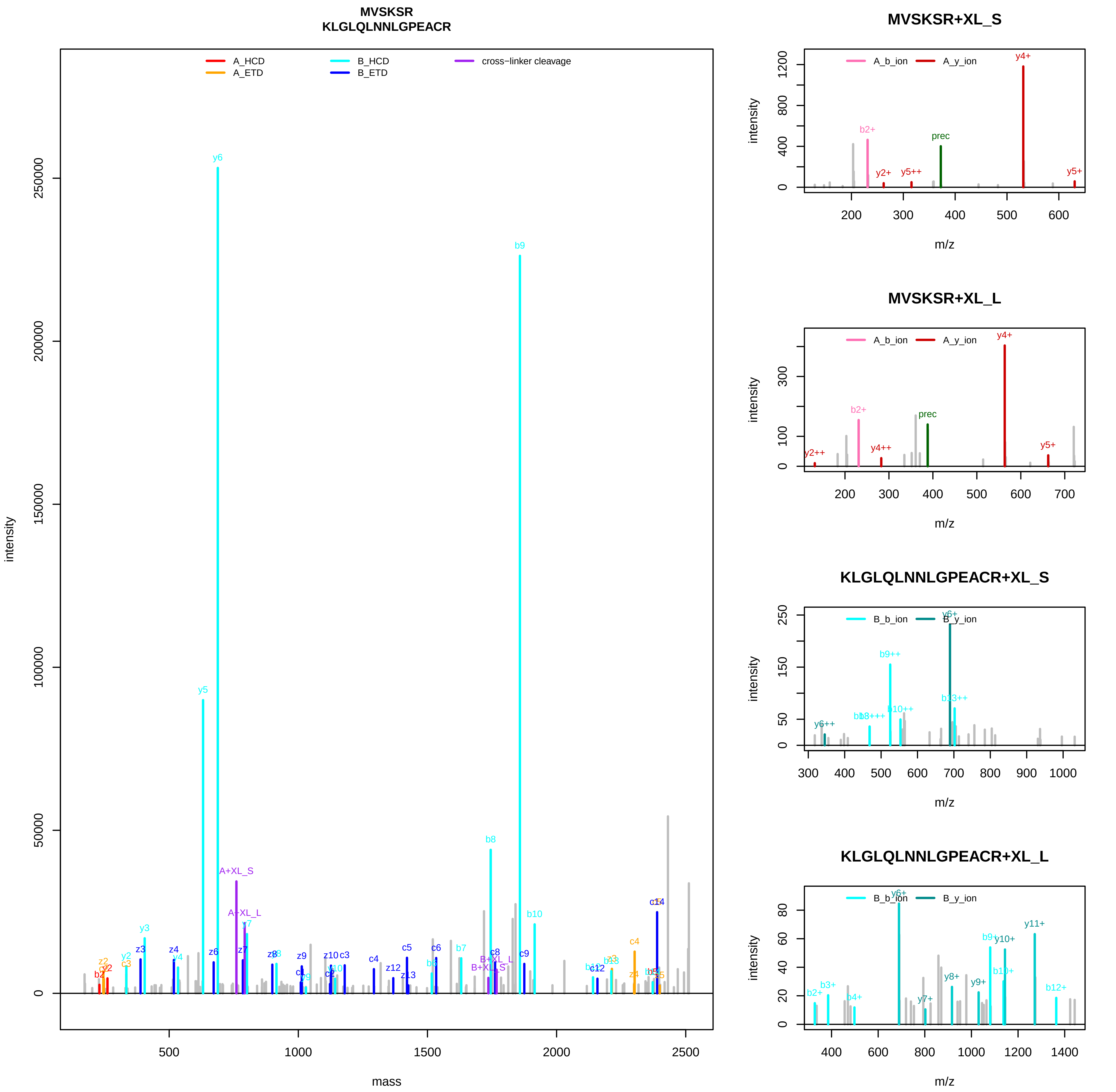
MVSKSR
KLGLQLNNLGPEACR
A_HCD
A_ETD
B_HCD
B_ETD
cross−linker cleavage
intensity
mass
y6
b9
y5
b8
A+XL_S
A+XL_L
y3
c14
b10
c4
MVSKSR+XL_S
A_b_ion
A_y_ion
y4+
b2+
prec
y2+
y5++
y5+
m/z
MVSKSR+XL_L
y2++
y4++
KLGLQLNNLGPEACR+XL_S
B_b_ion
B_y_ion
y6+
b9++
b13++
b10++
y6++
KLGLQLNNLGPEACR+XL_L
y11+
b9+
y10+
b10+
b12+
y8+
y9+
y7+
b2+
b3+
b4+
